# N6-methyladenosine methyltransferase METTL3 affects the phenotype of cerebral arteriovenous malformation via modulating Notch signaling pathway

**DOI:** 10.1186/s12929-020-00655-w

**Published:** 2020-05-09

**Authors:** Lin-jian Wang, Yimeng Xue, Ran Huo, Zihan Yan, Hongyuan Xu, Hao Li, Jia Wang, Qian Zhang, Yong Cao, Ji-zong Zhao

**Affiliations:** 1 Department of Neurosurgery, Beijing Tiantan Hospital, Capital Medical University, No.119 South 4th Ring West Road, Fengtai District, Beijing, 100070 China; 2 China National Clinical Research Center for Neurological Diseases, Beijing, China; 3 Savaid Medical School, University of Chinese Academy of Sciences, Beijing, 100049 China; 4 Center of Stroke, Beijing Institute for Brain Disorders, Beijing, China; 5 Beijing Key Laboratory of Translational Medicine for Cerebrovascular Disease, Beijing, China

**Keywords:** Cerebral arteriovenous malformation, METTL3, Nidus size, Notch signaling pathway, DTX3L, Angiogenesis

## Abstract

**Background:**

Cerebral arteriovenous malformation (AVM) is a serious life-threatening congenital cerebrovascular disease. Specific anatomical features, such as nidus size, location, and venous drainage, have been validated to affect treatment outcomes. Until recently, molecular biomarkers and corresponding molecular mechanism related to anatomical features and treatment outcomes remain unknown.

**Methods:**

RNA N6-methyladenosine (m^6^A) Methyltransferase METTL3 was identified as a differentially expressed gene in groups with different lesion sizes by analyzing the transcriptome sequencing (RNA-seq) data. Tube formation and wound healing assays were performed to investigate the effect of METTL3 on angiogenesis. In addition, Methylated RNA Immunoprecipitation Sequencing technology (MeRIP-seq) was performed to screen downstream targets of METTL3 in endothelial cells and to fully clarify the specific underlying molecular mechanisms affecting the phenotype of cerebral AVM.

**Results:**

In the current study, we found that the expression level of METTL3 was reduced in the larger pathological tissues of cerebral AVMs. Moreover, knockdown of METTL3 significantly affected angiogenesis of the human endothelial cells. Mechanistically, down-regulation of METTL3 reduced the level of heterodimeric Notch E3 ubiquitin ligase formed by DTX1 and DTX3L, thereby continuously activating the Notch signaling pathway. Ultimately, the up-regulated downstream genes of Notch signaling pathway dramatically affected the angiogenesis of endothelial cells. In addition, we demonstrated that blocking Notch pathway with DAPT could restore the phenotype of METTL3 deficient endothelial cells.

**Conclusions:**

Our findings revealed the mechanism by which m^6^A modification regulated the angiogenesis and might provide potential biomarkers to predict the outcome of treatment, as well as provide suitable pharmacological targets for preventing the formation and progression of cerebral AVM.

## Background

Cerebral arteriovenous malformation (AVM) is a fatal congenital vascular disease. Maldevelopment or lack of capillary network in the lesion regions leads to direct connection between cerebral arteries and veins, resulting in a series of brain hemodynamic disorders [1]. In addition to conservative treatment, microsurgical resection, embolization, stereotactic radiosurgery (SRS), or a combination of these modalities have been evolved to treat cerebral AVMs [2]. Specific anatomical features of cerebral AVMs form the basis of several commonly used grading scales that have been validated to predict treatment outcomes. The most widely used Spetzler-Martin grading of cerebral AVMs were categorized based on nidus size (maximum diameter, MD), location (in or not in the eloquent cortex, which refers to the regions that control motor, sensory, visual, and language functions), and the presence or absence of deep venous drainage. It can be used not only to predict the outcome of microsurgical treatment, but also to predict the SRS outcome [3, 4]. Among these anatomical features, it is particularly noteworthy that nidus size was also significantly associated with seizures and other clinical symptoms. However, genetic and molecular biomarkers associated with anatomical features and risks of treatment outcomes have not yet been identified, and a large amount of research is still needed to elucidate the progression and pathogenesis of cerebral AVM.

Recently, it is well established that N6-methyladenosine (m^6^A) is the most common and abundant RNA molecular modification in eukaryotes [5]. And the catalytic subunit METTL3 and METTL14, along with the regulate subunit WTAP and KIAA1429, form the core methyltransferase complex to catalyze the m^6^A modification of adenosine on RNA [6–10]. Accumulating evidences have shown that m^6^A modifications exert great influence on the multiple metabolic processes of RNA, such as shearing, nuclear transport, translation ability, stability, and transcription [11–19], and play critical roles in many bioprocesses, including circadian rhythm, DNA damage response, sex determination, developmental arrest, neuronal disorder, infectious diseases and tumorigenesis [20, 21]. Especially noteworthy is that METTL3 deficiency results in continuous activation of Notch signaling in dorsal aorta endothelial cells, eventually blocking the endothelial-to-haematopoietic transition [22]. In addition, Notch signaling pathway has complex and context-dependent effects on angiogenesis [23, 24], and has been reported to be activated in human cerebral AVMs [25, 26]. More interestingly, both gain and loss of Notch signaling can result in AVM formation [27–31]. However, it has not yet been determined whether METTL3 is implicated in the formation and progression of human cerebral AVMs by regulating m^6^A modification.

Here, we reported that the expression level of METTL3 was down-regulated in larger cerebral AVMs lesions. Moreover, knockdown of METTL3 significantly affected the tube formation and migration of endothelial cells, suggesting the properties of vascular endothelial cell can be modulated by m^6^A modification. Therefore, METTL3 might be used as a biomarker linked to the size of AVM lesions to predict the treatment outcomes. In order to fully clarify the specific underlying molecular mechanism, we performed RNA transcriptome sequencing (RNA-seq) and Methylated RNA Immunoprecipitation Sequencing technology (MeRIP-seq) to profile the changes of transcriptome-wide m^6^A modification sites and mRNA expression level in METTL3 deficient endothelial cells. Finally, we determined that DTX3L, the direct downstream target of METTL3, synergized with DTX1 to modulate the Notch signaling pathway to regulate angiogenesis in vitro. Our findings might contribute to understanding in more detail how arteriovenous malformations develop, and also might provide suitable targets for pharmacological treatment to prevent the progression and enlargement of cerebral AVMs in the future.

## Materials and methods

### Patients and samples

Detailed information on patient recruitment and sample preparation could be found in our previous study [32]. In Brief, cerebral AVMs samples were collected from consecutive patients undergoing surgical treatment. The clinical diagnosis of cerebral AVM was confirmed using digital subtraction angiography. The size and location (in or not in the eloquent cortex) of the lesion, the presence or absence of deep venous drainage and Spetzler-Martin grade were determined based on the imaging results. Finally, from September 2016 to November 2017, a total of 57 patients were enrolled in this study. Informed consents were obtained from all patients, and this study was approved by the institutional review board of Beijing Tiantan Hospital, Capital Medical University. The baseline characteristics of all patients are summarized in Table S1.

### Cell culture

The Human Umbilical Vein Endothelial Cells (HUVECs) were purchased from ScienCell (Carlsbad, CA), and cultured in Endothelial Cell Medium (ECM, ScienCell) supplemented with 5% fetal bovine serum (FBS, Gibco), 100 U/ml penicillin and 100 μg/ml streptomycin.

### Gene silencing

The siRNAs were designed and synthesized by RIBOBIO (Table S2). Endothelial cells were transfected with siRNAs using Lipofectamine RNAiMAX (Invitrogen). After transfection for 48 h, cells were harvested for subsequent mRNA or protein expression analysis.

### Plasmid construction and transfection

The plasmids expressing Flag-tagged *Homo sapiens* METTL3 or Flag-tagged *Homo sapiens* HEY2 were synthesized by Shanghai Genechem Co., Ltd. Constructed plasmids were transfected into the endothelial cells according to the manufacturer’s instructions of jetPRIME kit (Polyplus-transfection).

### RNA extraction, cDNA synthesis and quantitative real-time PCR (qRT-PCR)

Total RNA was extracted from endothelial cells according to the manufacturer’s instructions for TRIzol reagent (Invitrogen, USA) and then dissolved in RNase-free water. The cDNA was synthesized using PrimeScript™ RT reagent Kit with gDNA Eraser (TaKaRa Co. Dalian, China). Quantitative Real-Time PCR was performed using the SYBR® Premix Ex Taq™ II (Tli RNaseH Plus) (TaKaRa) on the QuantStudio™ real-time PCR system (Applied Biosystems, Foster City, CA, USA). The specific primers used in this paper were listed in Table S3.

### Immunoblotting

Cells were harvested and lysed in RIPA lysis buffer with protease and phosphatase inhibitors. Protein samples were separated by sodium dodecyl sulfate polyacrylamide gel electrophoresis (SDS-PAGE) and transferred to 0.45 μm polyvinylidene difluoride (PVDF) membrane (Merck Millipore). After blocking for 1 h in 5% bovine serum albumin (BSA), the membranes were incubated with the specific primary antibodies (Table S4). And then, the PVDF membrane was incubated with Horseradish peroxidase-conjugated secondary antibodies and immunoreactive bands were visualized with enhanced chemiluminescence reagent (Merck Millipore) according to the manufacturer’s instructions.

### Methylated RNA immunoprecipitation (MeRIP)

MeRIP were performing using Magna MeRIP m^6^A Kit (Millipore, 17–10,499) according to the manufacturer’s instructions. Briefly, 300 μg of total RNA was chemically fragmented into about 100 nucleotides in length by incubation at 94 °C for 1 min in fragmentation buffer, followed by magnetic immunoprecipitation with the monoclonal antibody toward m^6^A. Methylated RNA was eluted by competition with free m^6^A, and extracted with RNeasy kit (Qiagen). Both the IP samples and the input samples without immunoprecipitation were used for RNA-seq library generation with NEBNext® Ultra II Directional RNA Library Prep Kit (New England Biolabs, Inc., USA). Thereafter, sequencing was performed by Cloud-Seq Biotech Ltd. Co. (Shanghai, China). The raw data of this study have been deposited in GEO database (GSE142386). For m^6^A-IP-qPCR, total RNA was chemically fragmented into about 300 nucleotides in length. One-tenth of the fragmented RNA was saved as input control, and further analysed by qPCR along with eluted methylated RNA.

### mRNA stability assays

Control and genes silencing endothelial cells were treated with 10 μg per ml actinomycin D (MCE, HY-17559). The total RNAs were then extracted by TRIzol (Invitrogen) at indicated time points and analyzed by qRT-PCR. The turnover rate and half-life of mRNA was estimated according to a previously published paper [33].

### Cell migration

Cell migration experiments were conducted according to the manufacturer’s protocol, 70 μl cell suspension at the 5 × 10^5^ cells/ml concentration was seeding into the ibidi Culture-Insert 2 Well in μ-Dish 35 mm, which would result in a confluent layer within 24 h. After appropriate cell attachment (incubated at 37 °C and 5% CO_2_ for 24 h), the Culture-Insert 2 Well was gently removed by using sterile tweezer. Then, the used wells were filled with cell free medium, and the original images were obtained by using the Fluorescence Inversion Microscope System. 24 h later, the migrated images were acquired and analyzed with the original images by using the Image J software.

### Tube formation assays

Gel matrix was prepared according to the manufacturer’s protocol or reference. The inner well of μ-Slide was filled with 10 μl liquid gel, which could make the gel polymerize under appropriate conditions. Cell suspension at 3 × 10^5^ cells/ml concentration was prepared, and 50 μl of the cell suspension was applied into the upper well. Then the μ–Slide was covered with the supplied lid and incubated at 37 °C and 5% CO_2_ as usual. About 24 h later, the tube formation images were taken under the Fluorescence Inversion Microscope System and analyzed by using the Image J software.

### Statistical analysis

All experiments were performed at least three independent replicates and statistical analyses were performed with GraphPad Prism6 software. Statistical significance was calculated by two-way ANOVA, one-way ANOVA and/or unpaired Student’s t-test. A *p* value less than 0.05 was considered statistically significant.

## Results

### METTL3 is related to the nidus size of cerebral AVMs

A total of 57 patients with cerebral AVMs were included in this study. The baseline characteristics of all patients were summarized in Table S1. Specific anatomical features and Spetzler-Martin grade were determined according to the imaging results of digital subtraction angiography. There were 13 patients (22.8%) with deep vein drainage. The lesions of 25 patients (43.9%) were located in eloquent brain areas. The maximum diameter of lesions ranged from 1.1 cm to 5.9 cm with a mean of 3.3 cm. According to Spetzler-Martin grade, 18 patients (31.6%) were classified as grade 1, 16 patients (28.1%) were classified as grade 2, 15 patients (26.3%) were classified as grade 3, and 8 patients (14.0%) were classified as grade 4. In addition, 21 patients (36.8%) had intracranial hemorrhage.

**Fig. 1:**
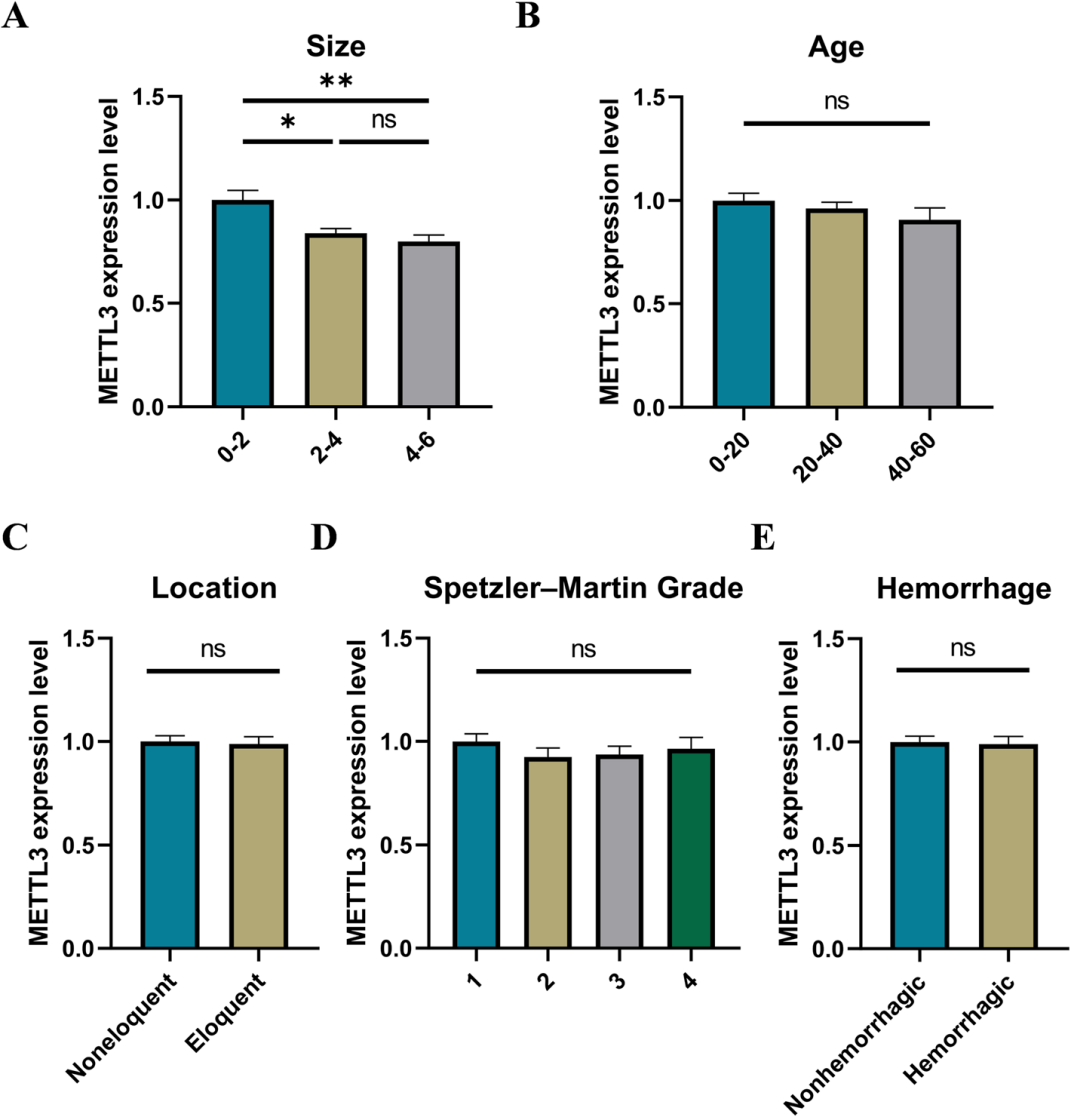
METTL3 is related to the nidus size of cerebral AVMs. **a** The expression levels of METTL3 in different size groups. All patients were classified into 3 groups according to maximum diameter, 0 cm < MD ≤ 2 cm, *n* = 5; 2 cm < MD ≤ 4 cm, *n* = 39; 4 cm < MD ≤ 6 cm, *n* = 13. **b** The expression levels of METTL3 in different age groups, 0 < age ≤ 20, *n* = 22; 20 < age ≤ 40, *n* = 23; 40 < age ≤ 60, *n* = 12 **c** The expression levels of METTL3 in different location groups. Eloquent, *n* = 25; Noneloquent, *n* = 32. **d** The expression levels of METTL3 in different Spetzler-Martin grading groups. Grade 1, *n* = 18; Grade 2, *n* = 16; Grade 3, *n* = 15; Grade 4, *n* = 8. **e** The expression levels of METTL3 in different hemorrhagic presentation groups. Hemorrhagic, *n* = 21; Non-hemorrhagic, *n* = 36. *P* values were calculated using one-way ANOVA and/or Student’s t-test. *, *P* < 0.05; **, *P* < 0.01; ***, *P* < 0.001

Previously, we have performed transcriptome sequencing and identified that WTAP was down-regulated in the cerebral AVMs lesions compared to the normal intracranial vascular tissues [32]. Here, we analyzed the differential expression profiles between specific groups of anatomical features, with particular attention to the expression of m^6^A-related molecules. Finally, we found that the METTL3 expression levels were reduced in the larger nidus compared to smaller nidus (Fig. 1a). Meanwhile, no significant differences were found between different hemorrhagic presentation, different locations, or different Spetzler-Martin grade groups (Fig. 1b-e). However, in the hemorrhagic group, we found that the METTL3 expression levels were not only related to the nidus size of cerebral AVMs, but also related to the age, locations, and Spetzler-Martin grade (Fig. S1). Therefore, METTL3 might be a biomarker related to the nidus size of cerebral AVMs and might be involved in the progression of cerebral AVM.

### METTL3 is required for angiogenesis of endothelial cells

**Fig. 2:**
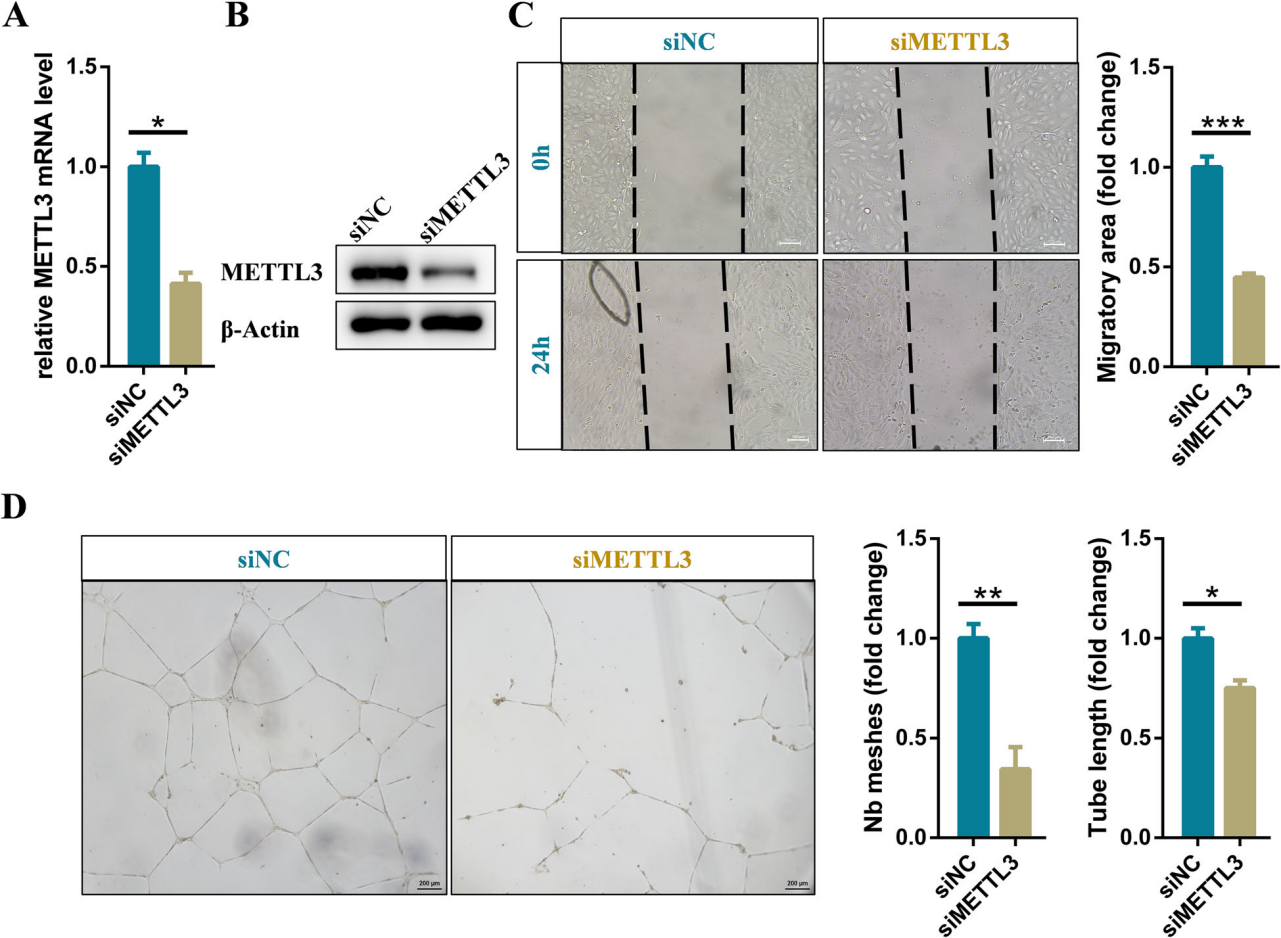
METTL3 is required for angiogenesis of human endothelial cells. **a** qRT-PCR and (**b**) western blot analysis of the knockdown efficiency of siRNA specifically target on METTL3 in endothelial cells. **c** Representative images and statistical analysis of cell migration assay for control and METTL3 deficient endothelial cells at the indicated times. **d** Representative bright-field images and statistical analysis of tube formation assay of control and METTL3 deficient endothelial cells. Data are shown as mean ± SEM of three independent experiments. *P* values were calculated using Student’s t-test. *, *P* < 0.05; **, *P* < 0.01; ***, *P* < 0.001

In cerebral AVMs, abnormalities in angiogenesis such as tube formation and migration of vascular endothelial cells have been reported. To fully determined the effects of METTL3 on these characteristics, we knocked down METTL3 in endothelial cells by transfecting specific siRNA, and verified knockdown efficiency with qRT-PCR and western blot (Fig. 2a and b). The data of wound healing and migration assays indicated the migratory ability of METTL3 deficient endothelial cells was evidently decreased (Fig. 2c). Moreover, the tube formation results showed that knockdown of METTL3 significantly inhibited formation of capillary-like structures (Fig. 2d). Overall, these results revealed that the expression level of METTL3 could significantly affect angiogenesis of vascular endothelial cells and might therefore affect the size of the lesions.

### Notch pathway is activated in METTL3 deficient endothelial cells

**Fig. 3:**
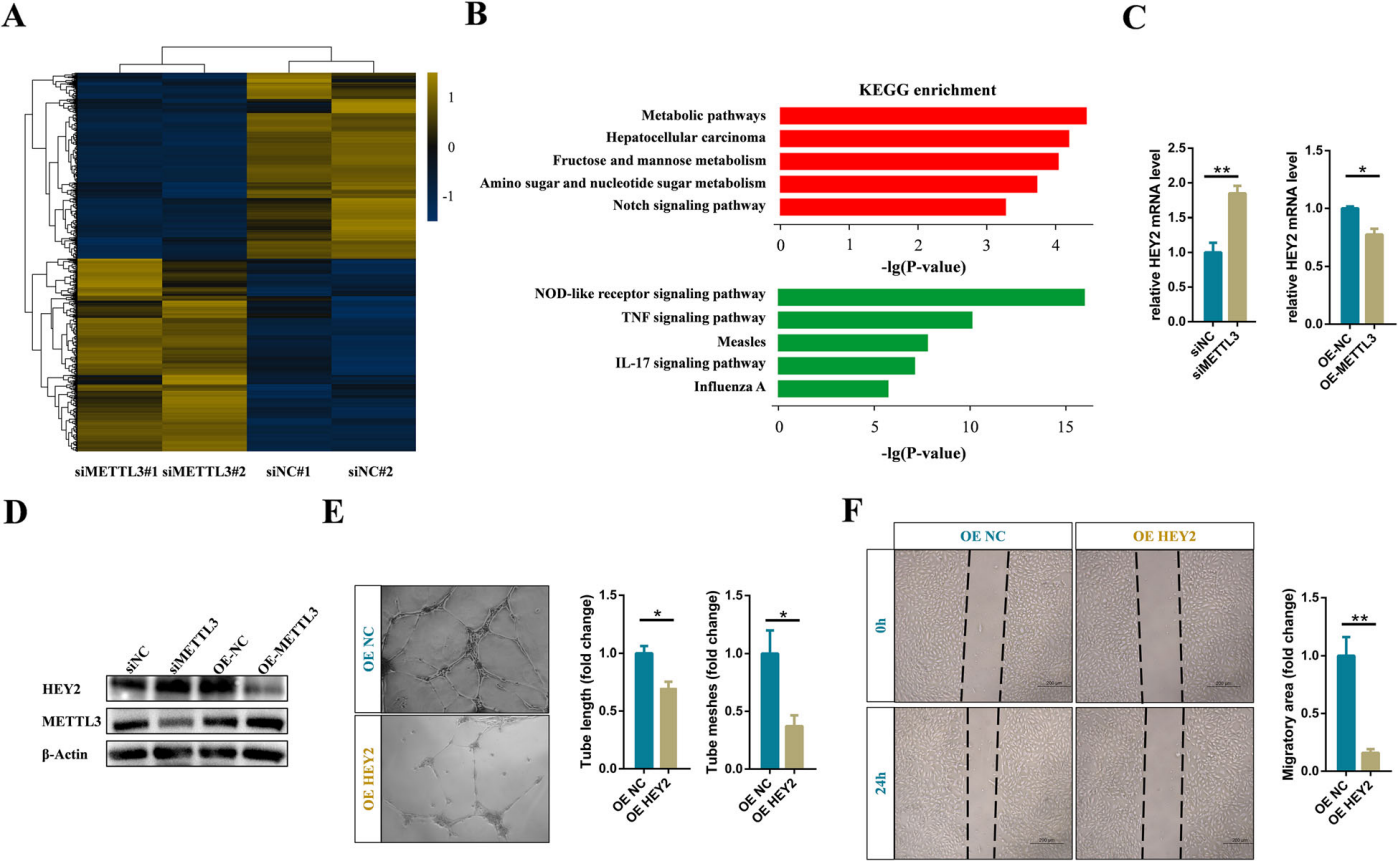
Notch pathway is activated in METTL3 deficient endothelial cells. **a** Heatmap depicting differentially expressed genes between control and METTL3 deficient endothelial cells (fold change > 1.2, *p*-value < 0.05). **b** KEGG analysis of the upregulated (upper) and downregulated (down) genes in METTL3 deficient endothelial cells. **c** qRT-PCR and (**d**) western blot analysis of the expression level of HEY2 after silencing or overexpressing of METTL3. **e** Representative bright-field images and statistical analysis of tube formation assay and (**f**) cell migration assay of control and HEY2 overexpressing cells. Data are shown as mean ± SEM of three independent experiments. *P* values were calculated using Student’s t-test. *, *P* < 0.05; **, *P* < 0.01; ***, *P* < 0.001

In order to identify the target genes involved in angiogenesis, we performed mRNA transcriptome sequencing to interrogate the expression changes between the control and METTL3 deficient endothelial cells. The data showed 706 and 680 genes were significantly up-regulated and down-regulated in METTL3 deficient endothelial cells, respectively (Fig. 3a). Intriguingly, similar to METTL3 deficiency in zebrafish embryos, pathway analysis showed that highly up-regulated genes in METTL3 deficient human endothelial cells also significantly enriched in the Notch signaling pathway (Fig. 3b). Notch is activated by a unique process that includes ligand binding and multistep proteolytic processing, which induces the transcription of Notch target genes, such as HES1, HEY1, and the like bHLH transcription factors [34, 35]. Naturally, we examined the expression of the Notch downstream genes in METTL3 deficient and overexpressing endothelial cells. The results showed that knockdown of METTL3 significantly up-regulated the mRNA and protein levels of the putative arterial endothelial marker HEY2 in endothelial cells (Fig. 3c and d). And, correspondingly, overexpression of METTL3 significantly decreased the expression level of HEY2 (Fig. 3c and d). Therefore, we concluded that the Notch signaling pathway was regulated by METTL3 in endothelial cells. Subsequently, we verified the effects of HEY2 on tube formation and migration in endothelial cells. Same as the phenotype of endothelial cells with knockdown of METTL3, overexpression of HEY2 significantly affected the formation of capillary-like tubes and migration of endothelial cells (Fig. 3e and f).

BMPs belong to the TGF-β superfamily of secreted growth factors and bind receptors to induce phosphorylation and nuclear translocation of SMAD transcription factors (SMAD1/5/9) and regulate vessel growth. However, previous study has proved that Notch directly regulates SMAD6 expression to affect BMP responsiveness of endothelial cells and new vessel branch formation [36]. Therefore, we also detected the expression level of SMAD6 in METTL3 deficient endothelial cells. Indeed, with the activation of the Notch signaling pathway, the expression level of SMAD6 also increased significantly in METTL3 deficient endothelial cells, while the phosphorylation modification of SMAD1/5/9 and SAMD2/3 significantly decreased (Fig. S2). Simultaneously, we found that overexpression of METTL3 suppressed the expression of SMAD6 and up-regulated BMP responsiveness (Fig. S2). Taken together, we confirmed that a decrease in METTL3 expression levels could activate the Notch pathway to affect angiogenesis in endothelial cells.

### METTL3 activates Notch pathway via reducing the expression of DTX3L in endothelial cells

**Fig. 4:**
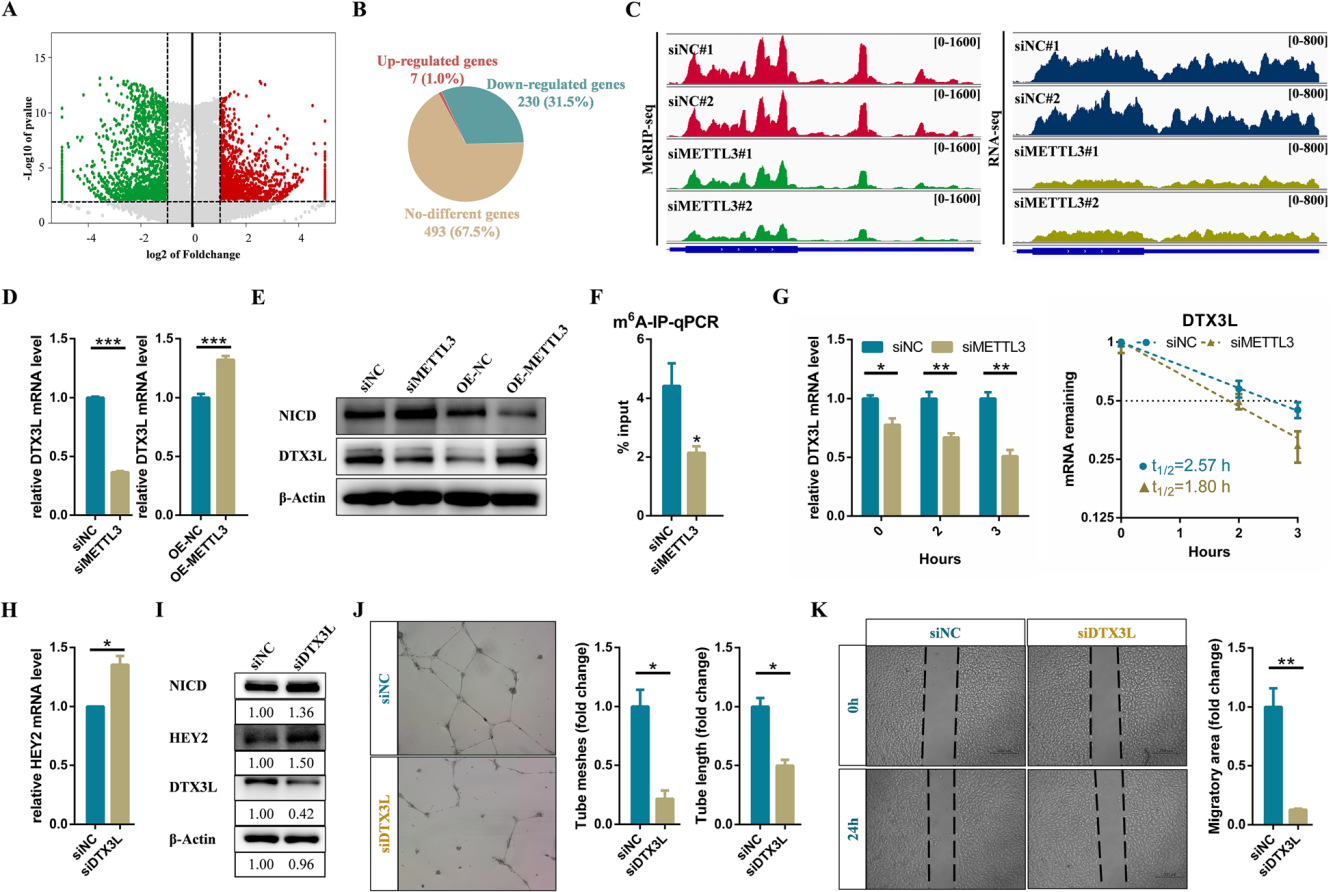
METTL3 activates Notch pathway via reducing the expression of DTX3L in endothelial cells. **a** Volcano map showing the m^6^A enrichment peaks in METTL3 deficient endothelial cells compared to control. Significantly increased and decreased peaks (fold change > 2, p-value < 0.001) are highlighted in red and green, respectively (**b**) Pie chart displaying the transcription level of genes with reduced m^6^A modification. **c** Integrative Genomics Viewer (IGV) tracks displaying MeRIP-seq and RNA-seq read distribution in DTX3L mRNA of control and METTL3 deficient cells. **d** qRT-PCR and (**e**) western blot analysis of the expression level of target genes. **f** m^6^A-IP-qPCR analysis of m^6^A enrichment on DTX3L mRNA in control and METTL3 deficient cells. **g** The mRNA half-life of DTX3L transcript in control and METTL3 depletion endothelial cells. **h** qRT-PCR and (**i**) Western blot analysis of indicated genes in control and DTX3L deficient endothelial cells. **j** Effects of DTX3L on tube formation and (**k**) migration of endothelial cells. Data are shown as mean ± SEM of three independent experiments. *P* values were calculated using Student’s t-test. *, *P* < 0.05; **, *P* < 0.01; ***, *P* < 0.001. *P* < 0.001

METTL3 is well known to be the most important m^6^A methyltransferase. Knockdown of METTL3 by specific siRNA significantly reduced the m^6^A modification level of RNAs in endothelial cells (Fig. S3A). To further clarify the downstream targets of METTL3, we performed Methylated RNA Immunoprecipitation Sequencing technology (MeRIP-seq) to profile the changes of transcriptome-wide m^6^A modification sites between METTL3 deficient and control endothelial cells. Finally, 1576 m^6^A modified transcripts were identified (Fig. 4a). Among them, 730 genes with significantly decreased m^6^A modification were identified as the potential targets of METTL3 (Fig. 4a). Pathway analysis revealed that the low levels of m^6^A modified transcripts were enriched in many biological processes such as MAPK signaling pathway, cAMP signaling pathway, and other immune-related signaling pathways (Fig. S3B). Previous study has showed that Notch1a is a directed target of METTL3 [22]. Surprisingly, although Notch1 were significantly up-regulated and down-regulated in METTL3 deficient and overexpressing endothelial cells, respectively, knockdown of METTL3 did not result in reduction of the m^6^A modification in Notch1 mRNA (Fig. S3C and D). In addition, knockdown of YTHDF2 did not up-regulated the mRNA and protein levels of Notch1 in human endothelial cells (Fig. S3E and F). A hypothesis raised by these results was that METTL3 could active the Notch pathway by regulating another molecular via m^6^A modification, rather than directly modulating Notch1 expression.

Then, we further analysed the expression levels of 730 potential targets with reduced m^6^A modification in METTL3 deficient endothelial cells. Two hundred thirty seven targets were shown to be differentially expressed genes, including 230 significantly down-regulated and 7 up-regulated genes (Fig. 4b). Previous study has demonstrated that DTX1 and DTX3L, functioning as a heterodimeric Notch E3 ligase, concertedly down-regulate Notch activity [37]. Interestingly, MeRIP-seq and RNA-seq results showed that the m^6^A modification and expression levels of DTX3L mRNA were decreased dramatically in METTL3 deficient endothelial cells (Fig. 4c). Moreover, our results of qRT-PCR and western blot showed that mRNA and protein levels of DTX3L were significantly down-regulated and up-regulated in METTL3 deficient and over-expressed endothelial cells (Fig. 4d and e). m^6^A-IP-qPCR assays also confirmed that DTX3L mRNA exhibited noticeably decreased m^6^A modification level in METTL3 deficient endothelial cells (Fig. 4f). Furthermore, knockdown of METTL3 markedly shortened half-live of DTX3L transcript, indicating that METTL3 regulated DTX3L expression through modulating mRNA stability (Fig. 4g). Therefore, we identified that DTX3L was a direct downstream target of METTL3.

To investigate whether DTX3L affected the Notch signaling pathway, we then knocked it down in endothelial cells. Finally, we found that the endogenous NICD level and the expression level of HEY2 were both up-regulated in DTX3L deficient endothelial cells (Fig. 4h and i). In addition, the endogenous NICD level was increased or decreased in METTL3 deficient or overexpressing endothelial cells (Fig. 4e). Therefore, these results suggested that METTL3 could activate the Notch pathway by m^6^A-dependent regulating its downstream target DTX3L. Tube formation and wound healing assays also showed that knockdown of DTX3L significantly affected angiogenesis of endothelial cells (Fig. 4j and k). In addition to DTX3L, the expression level of DTX1 was also decreased or increased in METTL3 deficient or overexpressing endothelial cells, respectively (Fig. S4). Previously, DTX3L and the human family of DTX proteins (DTX1, DTX2, and DTX3) have been found to function as E3 ligases based on their capacity for self-ubiquitination, while heterodimerization of DTX3L and DTXs enhances this activity [38]. Correspondingly, we speculated that down-regulated METTL3 in cerebral AVMs attenuated DTX3L and DTX1 to synergistically activate Notch signaling pathway, ultimately affecting angiogenesis of endothelial cells.

### IGF2BPs enhances DTX3L mRNA stability via an m^6^A-dependent manner

**Fig. 5:**
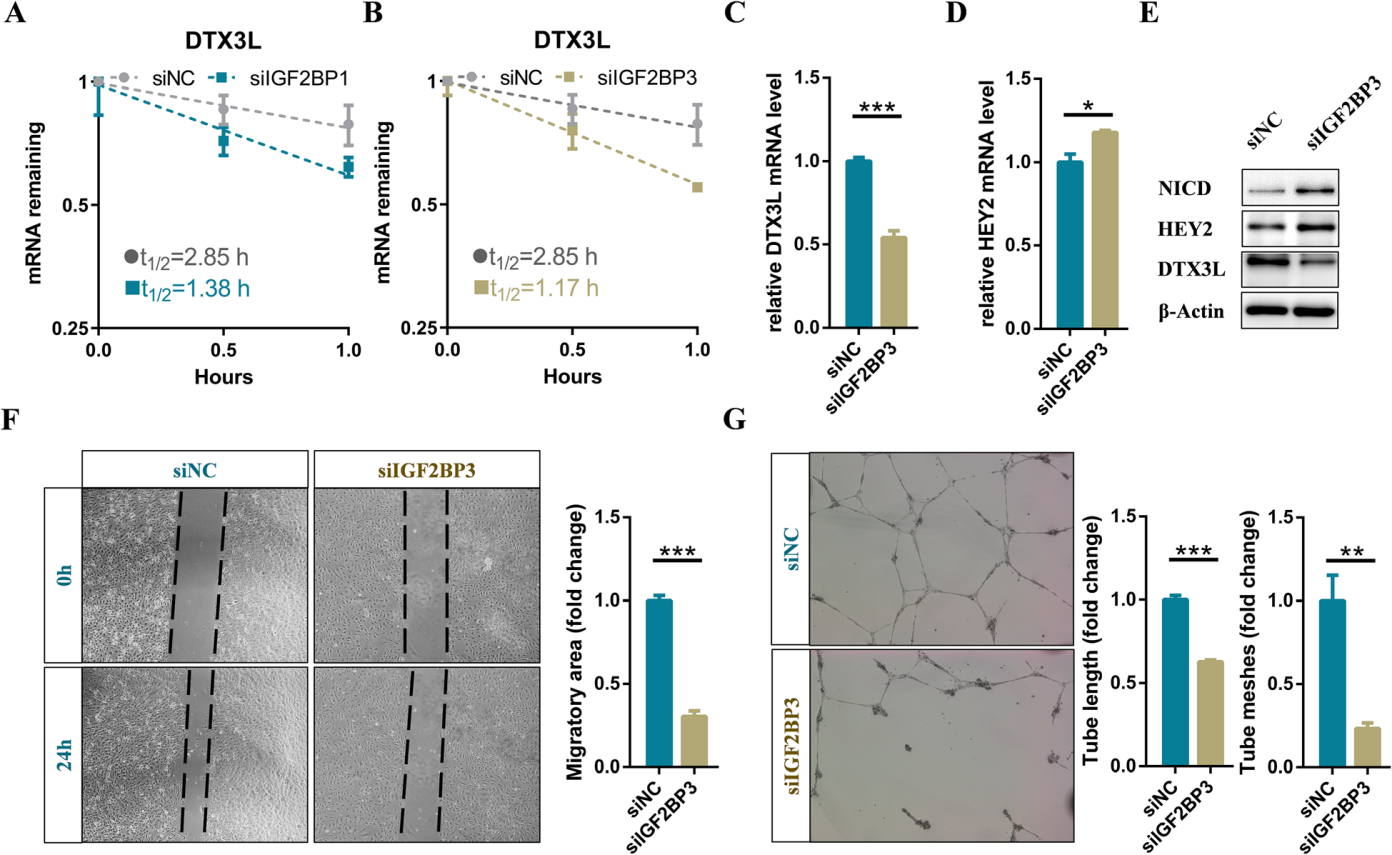
IGF2BPs enhances DTX3L mRNA stability via an m^6^A-dependent manner. **a** The mRNA half-life of DTX3L transcript in IGF2BP1 and (**b**) IGF2BP3 depletion endothelial cells, respectively. **c-d**) qRT-PCR and (**e**) western blot analysis of the expression level of specific genes in control and IGF2BP3 deficient cells. **f** Effects of IGF2BP3 on migration and (**g**) tube formation of endothelial cells. Data are shown as mean ± SEM of three independent experiments. *P* values were calculated using Student’s t-test. *, *P* < 0.05; **, *P* < 0.01; ***, *P* < 0.001

Previous studies have identified the IGF2BP family members, as m^6^A readers, promote the stability of their target mRNAs in an m^6^A-dependent manner [39, 40]. To elucidate whether IGF2BPs involved in the regulation of DTX3L, IGF2BP1, IGF2BP2 and IGF2BP3 were knocked down in endothelial cells, respectively. The RNA stability assays showed that the DTX3L mRNA half-lives were significantly affected by the inhibition of IGF2BP1 and IGF2BP3 (Fig. 5a and b). Owing to knockdown of IGF2BP3 mostly shortened the DTX3L mRNA half-lives, we choose it for further analysis (Fig. 5b). As expected, the mRNA and protein levels of DTX3L decreased dramatically after interfering the expression of IGF2BP3 in endothelial cells (Fig. 5c and e). Therefore, these results suggested that DTX3L was regulated via an m^6^A-IGF2BPs-dependent manner. Moreover, the endogenous NICD level and the expression level of HEY2 were up-regulated in IGF2BP3 deficient endothelial cells (Fig. 5d and e). Functionally, interference of IGF2BP3 significantly affected the tube formation and migration of endothelial cells (Fig. 5f and g). In summary, we concluded that METTL3 modulated the expression level of DTX3L depending on IGF2BPs.

### Notch pathway inhibitor DAPT rescues defective angiogenesis caused by knockdown of METTL3

**Fig. 6:**
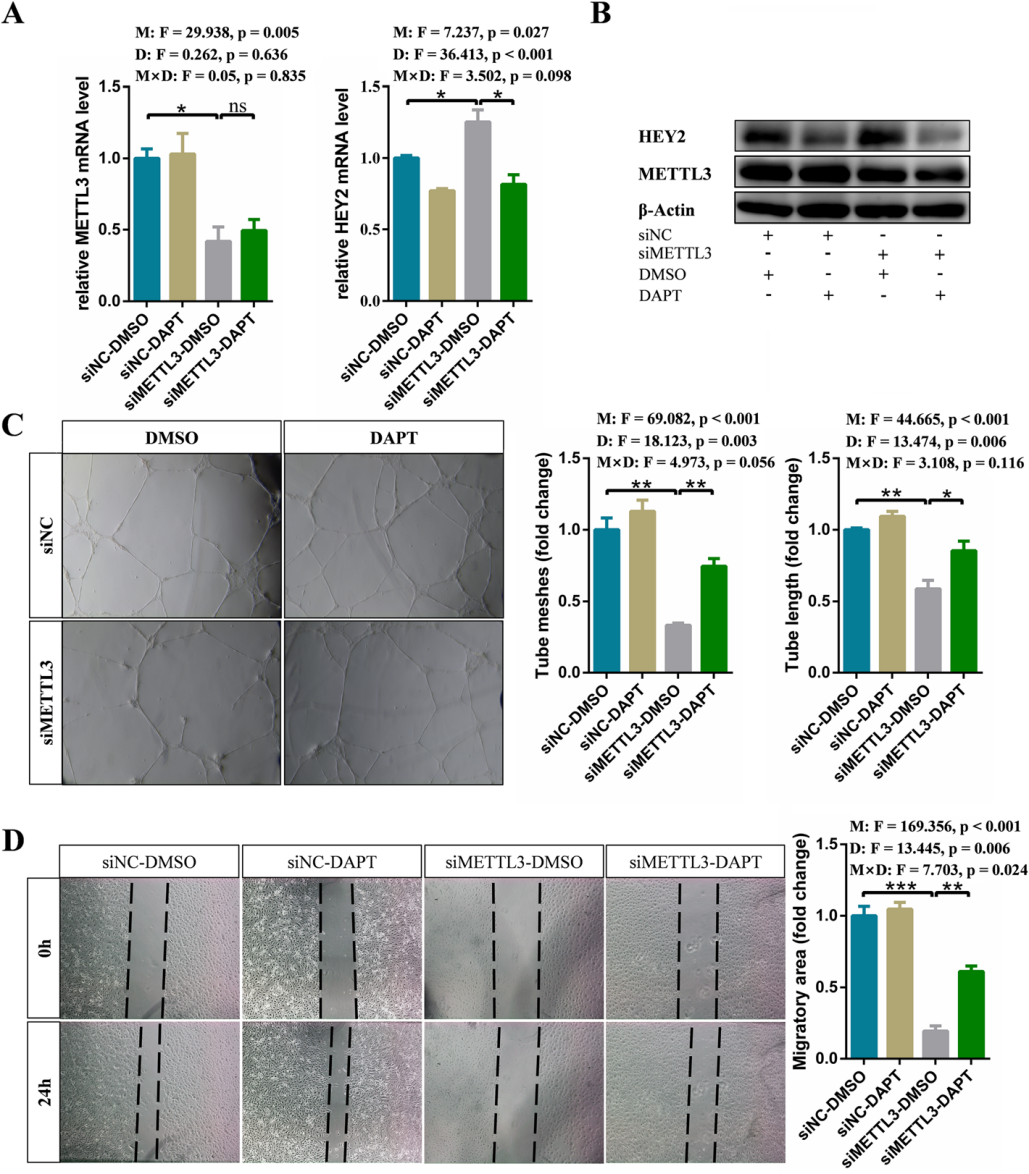
Notch pathway inhibitor DAPT rescues defective angiogenesis caused by knockdown of METTL3. **a** qRT-PCR and (**b**) western blot analysis of expression of target genes in control and METTL3 deficient endothelial cells which were treated with DMSO or DAPT, respectively. **c** Tube formation and (**g**) migration assay of control and METTL3 depletion cells after DMSO or DAPT treatment, respectively. Data are shown as mean ± SEM of three independent experiments. *P* values were calculated using two-way ANOVA followed by Student’s t-test. M, METTL3; D, DAPT; M × D, METTL3 × DAPT. *, *P* < 0.05; **, *P* < 0.01; ***, *P* < 0.001

Considering that the γ-secretase inhibitor DAPT can suppress Notch signaling pathway, we hypothesized that blocking Notch pathway with DAPT could restore the phenotype of METTL3 deficient endothelial cells. To address this hypothesis, the control and METTL3 deficient endothelial cells were treaded with DMSO and DAPT, respectively. As we speculated, compared to DMSO treatment, inhibition of Notch signaling by DAPT significantly reduced the mRNA and protein level of HEY2 in METTL3 deficient endothelial cells (Fig. 6a and b). Additionally, DAPT treatment enhanced tube formation and migration of METTL3 deficient endothelial cells (Fig. 6c and d). Therefore, DAPT may be an important potential therapeutic intervention for cerebral AVMs.

## Discussion

The clinical symptoms such as cerebral hemorrhage, epilepsy, and stroke, caused by cerebral AVM, seriously threaten the life safety of patients. Currently, the pathogenesis of cerebral arteriovenous malformation remains unclear. It is well known epigenetic mechanisms, including DNA, chromatin and histone modifications, play an important role in regulating gene expression [41]. More recently, emerging epitranscriptomics have revealed the extremely important role of RNA modifications in shaping the transcriptomic landscapes [42, 43]. In particular, m^6^A modifications are dynamic and reversible in mammalian cells, regulating all stages of the RNA life cycle [20]. Abnormal m^6^A modification levels lead to RNA metabolic disorders and contribute to the development of diseases [44]. Surprisingly, in this study, we found that the METTL3 expression levels were related to the nidus size of cerebral AVMs (Fig. 1a). In addition, depletion of METTL3 resulted in significantly lower m^6^A modification levels in RNA and affected angiogenesis of endothelial cells (Fig. 2c, d, and S3A). Therefore, m^6^A modification on RNA, which have been proposed as another layer of epigenetic regulation similar to DNA and histone methylation, may be associated with the development of cerebral AVMs.

Due to the lack of an intervening capillary bed, cerebral arteries and veins are directly connected, leading to hemodynamic changes in cerebral AVMs [45]. In addition, abnormal angiogenesis usually results in structural and functional defects in new blood vessels [46]. Typical angioarchitectural features, hemodynamic alterations and abnormal angiogenesis may result in disordered vascular hyperplasia in cerebral AVMs. Abnormal tangles of dilated vascular structures, including tortuous arteries and dilated veins, form nidus in cerebral AVMs. Therefore, disregulated angiogenesis may affect the size of the lesions in cerebral AVMs. The most widely used Spetzler-Martin grading of cerebral AVMs can be used not only to predict the outcome of microsurgical treatment, but also to predict the SRS outcome [3, 4]. Excellent results were seen with the resection of small AVMs in noneloquent brain with superficial venous drainage. Damage to the regions that control motor, sensory, visual, and language functions (termed eloquent areas of the cortex), as well as impairing deep white-matter pathways and basal ganglia structures are associated with a poor clinical outcome [4]. Larger cerebral AVMs surrounded by a larger volume of parenchyma may result in a higher likelihood of affecting these zones. Taking these into account, METTL3 might be identified as a molecular biomarker linked to treatment outcomes. Moreover, a better understanding of how METTL3 affects lesion size will help elucidate the progression of cerebral AVMs.

Similar to METTL3 deficiency in zebrafish embryos [22], knockdown of METTL3 significantly activated the Notch pathway in endothelial cells (Fig. 3a and b). However, m^6^A modifications on Notch1 mRNA were not altered in METTL3 deficient endothelial cells (Fig. S3C). Intriguingly, DTX1 and DTX3L, which synergistically blocked Notch1 activity through functioning as a heterodimeric Notch E3 ligase [37], were down-regulated in the METTL3 deficient endothelial cells (Fig. 4d, e, and S4). Moreover, DTX3L was identified as the directed downstream gene of METTL3 and was maintained by an m^6^A-IGF2BPs-dependent mechanism in endothelial cells (Fig. 4 and Fig. 5). Thus, Notch pathway was continuously activated due to the inhibition of ubiquitination degradation, suggesting another layer of dysregulation of Notch signaling that may be involved in cerebral AVMs.

The TGF-β superfamily, consisting of various soluble ligands and their cognate membrane-bound receptors, plays an important role in a diverse range of processes during vessel development [47]. The cross-talk between Notch signaling pathway and BMP pathway has been shown to modulate endothelial cell behavior during vascular development. For example, HEY2 acts as a downstream of Notch to repress BMP2 expression [48–50]. However, along with Notch activation, the up-regulated target HEY2 did not inhibited BMP2 in METTL3 deficient endothelial cells (data not shown). Multiple lines of evidences suggest that HEY proteins primarily act as direct transcriptional repressors. Therefore, additional targets of HEY2 might be involved in the regulation of angiogenesis in endothelial cells. Moreover, Notch can directly regulate transcription of SMAD6 to repress BMP pathway responsiveness [36], which was also confirmed in this study (Fig. S2). Silencing of METTL3 dramatically up-regulated the expression of SMAD6 and inhibited the phosphorylation modification of the SMAD1/5/9 and SMAD2/3 (Fig. S2B). These findings suggested that METTL3 could affect angiogenesis of endothelial cells by negatively regulating TGF-β signaling pathway.

Except for anticonvulsants or acute symptomatic treatment to control associated seizures, so far, there are no drugs available to prevent bleeding or to effectively treat cerebral AVMs. Therefore, it is necessary to reveal the underlying mechanisms of cerebral AVMs and develop drugs accordingly. In this study, we found that down-regulated METTL3 could up-regulate Notch signaling pathway activity in cerebral AVMs. Moreover, Notch signaling pathway had also been shown to be abnormally activated in cerebral AVMs [25], so blocking Notch signaling may be a potential therapeutic pathway. Here, we demonstrated that DAPT could restore angiogenesis defects caused by reduced METTL3 expression levels and subsequent continuous activation of Notch signaling. Therefore, our results showed the potential therapeutic value of DAPT, and might lay a solid foundation for pharmacological treatment of cerebral AVMs.

## Conclusions

Collectively, our work suggests that METTL3 may be a potential molecular biomarker related to nidus size of cerebral AVMs and is likely to be used to predict the outcomes of treatment. Moreover, our results reveal a new paradigm for m^6^A-Notch axis regulating angiogenesis in cerebral AVM, which will provide suitable pharmacological targets for more effective treatment of patients.

## Supplementary information

**Additional file 1: Table S1.** Baseline characteristics of the samples.

**Additional file 2: Table S2.** siRNA used in this paper.

**Additional file 3 Table S3.** Primers used in this paper.

**Additional file 4: Table S4.** Antibody used in this paper.

**Additional file 5: Figure S1.** The expression levels of METTL3 in different groups of bleeding cerebral AVMs. (A) The expression levels of METTL3 in different size groups of bleeding cerebral AVMs. Small, 0 cm < MD ≤ 3 cm, *n* = 10; Medium, 3 cm < MD ≤ 6 cm, *n* = 11. (B) The expression levels of METTL3 in different age groups of bleeding cerebral AVMs. 0 < age ≤ 20, n = 10; 20 < age ≤ 40, *n* = 7; 40 < age ≤ 60, *n* = 4. (C) The expression levels of METTL3 in different location groups of bleeding cerebral AVMs. Eloquent, n = 11; Noneloquent, *n* = 10. (D) The expression levels of METTL3 in different Spetzler-Martin grading groups of bleeding cerebral AVMs. Grade 1, n = 7; Grade 2, *n* = 3; Grade 3, n = 7; Grade 4, n = 4. *P* values were calculated using one-way ANOVA and/or Student’s t-test. *, *P* < 0.05; **, *P* < 0.01; ***, *P* < 0.001.

**Additional file 6: Figure S2.** SMAD6 is up-regulated in METTL3 deficient endothelial cells. (A) qRT-PCR and (B) western blot analysis of the expression level of indicated genes in METTL3 silencing cells. Data are shown as mean ± SEM of three independent experiments. *P* values were calculated using Student’s t-test. *, *P* < 0.05; **, *P* < 0.01; ***, *P* < 0.001.

**Additional file 7: Figure S3.** Knockdown of METTL3 does not reduce m^6^A enrichment in Notch1 mRNA. (A) Dot blot showing the m^6^A modification level in control and METTL3 deficient endothelial cells. (B) GO analysis of the low m^6^A modification genes in METTL3 knockdown endothelial cells. (C) Integrative Genomics Viewer (IGV) tracks displaying MeRIP-seq read distribution in Notch1 mRNA of control and METTL3 knockdown endothelial cells. (D) qRT-PCR analysis of the expression level of Notch1 after silencing or overexpressing METTL3. (E) qRT-PCR and (F) western blot analysis of the expression level of Notch1 in YTHDF2 silencing cells. Data are shown as mean ± SEM of three independent experiments. *P* values were calculated using Student’s t-test. *, *P* < 0.05; **, *P* < 0.01; ***, *P* < 0.001.

**Additional file 8: Figure S4.** DTX1 is regulated by METTL3 in endothelial cells. (A) qRT-PCR and (B) western blot analysis of the expression level of DTX1 in METTL3 knockdown endothelial cells. Data are shown as mean ± SEM of three independent experiments. *P* values were calculated using Student’s t-test. *, *P* < 0.05; **, *P* < 0.01; ***, *P* < 0.001.

## Data Availability

The datasets used and analysed during the current study are available from the corresponding author on reasonable request.

## Abbreviations

AVM: Arteriovenous malformation
m^6^A: N6-methyladenosine
SRS: Stereotactic radiosurgery
qRT-PCR: quantitative Real-Time PCR
RNA-seq: RNA transcriptome sequencing
MeRIP-seq: Methylated RNA Immunoprecipitation sequencing
HUVEC: Human Umbilical Vein Endothelial Cell
