# Supplementary material for: N6-methyladenosine methyltransferase METTL3 affects the phenotype of cerebral arteriovenous malformation via modulating Notch signaling pathway
Source: J Biomed Sci. 2020 May 9;27:62. doi: 10.1186/s12929-020-00655-w (PMC7210675; doi:10.1186/s12929-020-00655-w)
Supplement: Supplementary file 1 — Additional file 1: Table S1. Baseline characteristics of the samples. [file 12929_2020_655_MOESM1_ESM.docx]

**Table s1.** Baseline characteristics of the samples.

| Sex | male | | | female | | |
| --- | --- | --- | --- | --- | --- | --- |
|  | 34 (59.6%) | | | 23 (40.4%) | | |
| Age | 28.5 (6-59) | | | | | |
| Seizure | Yes | | | No | | |
|  | 23 (40.4%) | | | 35 (59.6%) | | |
| Hemorrhage | Yes | | | No | | |
|  | 21 (36.8%) | | | 36 (63.2%) | | |
| Location | Eloquent | | | Noneloquent | | |
|  | 25 (43.9%) | | | 32 (56.1%) | | |
| Veins | Deep | | | Superficial | | |
|  | 13 (22.8%) | | | 44 (77.2%) | | |
| Size | 0-2 cm | | 2-4 cm | | 4-6 cm | |
|  | 5 (8.8%) | | 39 (68.4%) | | 13 (22.8%) | |
| Spetzler-Martin | 1 | 2 | | 3 | | 4 |
|  | 18 (31.6%) | 16 (28.1%) | | 15 (26.3%) | | 8 (14.0%) |
