# Supplementary material for: N6-methyladenosine methyltransferase METTL3 affects the phenotype of cerebral arteriovenous malformation via modulating Notch signaling pathway
Source: J Biomed Sci. 2020 May 9;27:62. doi: 10.1186/s12929-020-00655-w (PMC7210675; doi:10.1186/s12929-020-00655-w)
Supplement: Supplementary file 2 — Additional file 2: Table S2. siRNA used in this paper. [file 12929_2020_655_MOESM2_ESM.docx]

| **Table 2. siRNA used in this paper** | | |
| --- | --- | --- |
| **Targets** | **Sequences (5'-3')** | |
| siMETTL3 | #1 | GCACTTGGATCTACGGAAT |
|  | #2 | CGACTACAGTAGCTGCCTT |
|  | #3 | CAGTGGATCTGTTGTGATA |
| siDTX3L | #1 | GCACCATTGTGATTACTTA |
|  | #2 | GCAGCAAGGTTTCTGAGAA |
|  | #3 | GAAACACCGTCTGGTGATA |
| siDTX1 | #1 | GACGCTAGCTACCTAGACA |
|  | #2 | CGAGGATGTGGTTCGAAGA |
|  | #3 | CCATCCGCATCGTCTATGA |
| siIGF2BP1 | #1 | GGCTCAGTATGGTACAGTA |
|  | #2 | TGAAGATCCTGGCCCATAA |
|  | #3 | GAAGGACGGAACCTGAAGA |
| siIGF2BP2 | #1 | CATGCCGCATGATTCTTGA |
|  | #2 | GAACGAACTGCAGAACTTA |
|  | #3 | AACAGGGACCAAGATAACA |
| siIGF2BP3 | #1 | GCTGAGAAGTCGATTACTA |
|  | #2 | TAAGGAAGCTCAAGATATA |
|  | #3 | TCGGAAACTTCAGATACGA |
| siYTHDF2 | # | AAGGACGUUCCCAAUAGCCAA |
| siNC | # | UUCUCCGACGUGUCACGU |
