# Supplementary material for: N6-methyladenosine methyltransferase METTL3 affects the phenotype of cerebral arteriovenous malformation via modulating Notch signaling pathway
Source: J Biomed Sci. 2020 May 9;27:62. doi: 10.1186/s12929-020-00655-w (PMC7210675; doi:10.1186/s12929-020-00655-w)
Supplement: Supplementary file 3 — Additional file 3 Table S3. Primers used in this paper. [file 12929_2020_655_MOESM3_ESM.docx]

| **Table s3. Primers used in this paper** | | |
| --- | --- | --- |
| **Genes** | **Sequences (5'-3')** | |
| METTL3 | Forward | ATCCCCAAGGCTTCAACCAG |
|  | Reverse | GCGAGTGCCAGGAGATAGTC |
| NOTCH1 | Forward | TGCCTGGACAAGATCAATGAG |
|  | Reverse | CAGGTGTAAGTGTTGGGTCC |
| HEY2 | Forward | ATTATAGAGAAAAGGCGTCGGG |
|  | Reverse | GCATCTTCAAATGATCCACTGTC |
| TGFBR3 | Forward | CGGAAACATCACCTTCAACATG |
|  | Reverse | CCCAGTTCTTGTTCAGCCTTAG |
| DTX3L | Forward | AAAGGAAATCAGCCAGAGGG |
|  | Reverse | GGGTATCTCTTTCCTGGGTTTG |
| DTX1 | Forward | AGTTCACCGCAAGAGGATTC |
|  | Reverse | GTGCCGATAGTGAAGATGAGTC |
| YTHDF2 | Forward | TAGCCAACTGCGACACATTC |
|  | Reverse | CACGACCTTGACGTTCCTTT |
| IGF2BP1 | Forward | AGATGGTGCAGGTGTTTATCC |
|  | Reverse | TTTGGAGTCAGGTGTTTCGG |
| IGF2BP2 | Forward | AATCTCTTCATCCCAACCCAG |
|  | Reverse | ATGACCATCCTTTCGCTGAC |
| IGF2BP3 | Forward | GTTTATCCCAGCTCTATCAGTCG |
|  | Reverse | TCACCATCCTCACTTTAGCATC |
| GAPDH | Forward | AATGACCCCTTCATTGAC |
|  | Reverse | TCCACGACGTACTCAGCGC |
