# Supplementary material for: N6-methyladenosine methyltransferase METTL3 affects the phenotype of cerebral arteriovenous malformation via modulating Notch signaling pathway
Source: J Biomed Sci. 2020 May 9;27:62. doi: 10.1186/s12929-020-00655-w (PMC7210675; doi:10.1186/s12929-020-00655-w)
Supplement: Supplementary file 4 — Additional file 4: Table S4. Antibody used in this paper. [file 12929_2020_655_MOESM4_ESM.docx]

| **Table s4. Antibodies used in this paper** | | |
| --- | --- | --- |
| **Antibodies** | **Source** | **Identifier** |
| m6A (N6-methyladenosine) antibody | Synaptic Systems | Cat# 202003 |
| Anti-NICD Antibody | abcam | Cat# ab83232 |
| Anti-METTL3 Antibody | abcam | Cat# ab195352 |
| Anti-HEY2 Antibody | abcam | Cat# ab167280 |
| Anti-NOTCH1 Antibody | proteintech | Cat# 20687-1-AP |
| Anti-DTX3L Antibody | proteintech | Cat# 11963-1-AP |
| Anti-DTX1 Antibody | proteintech | Cat# 18350-1-AP |
| Anti-TGFBR3 Antibody | proteintech | Cat# 20000-1-AP |
| Anti-p-SAMD1/5/9 Antibody | Cell Signaling Technology | Cat# 13820 |
| Anti-p-SAMD2 Antibody | Cell Signaling Technology | Cat# 3108 |
| Anti-SAMD6 Antibody | ABGENT | Cat# AP5344c |
| Anti-β-Actin Antibody | abcam | Cat# ab8227 |
