# Supplementary figures and images for: N6-methyladenosine methyltransferase METTL3 affects the phenotype of cerebral arteriovenous malformation via modulating Notch signaling pathway
Source: J Biomed Sci. 2020 May 9;27:62. doi: 10.1186/s12929-020-00655-w (PMC7210675; doi:10.1186/s12929-020-00655-w)

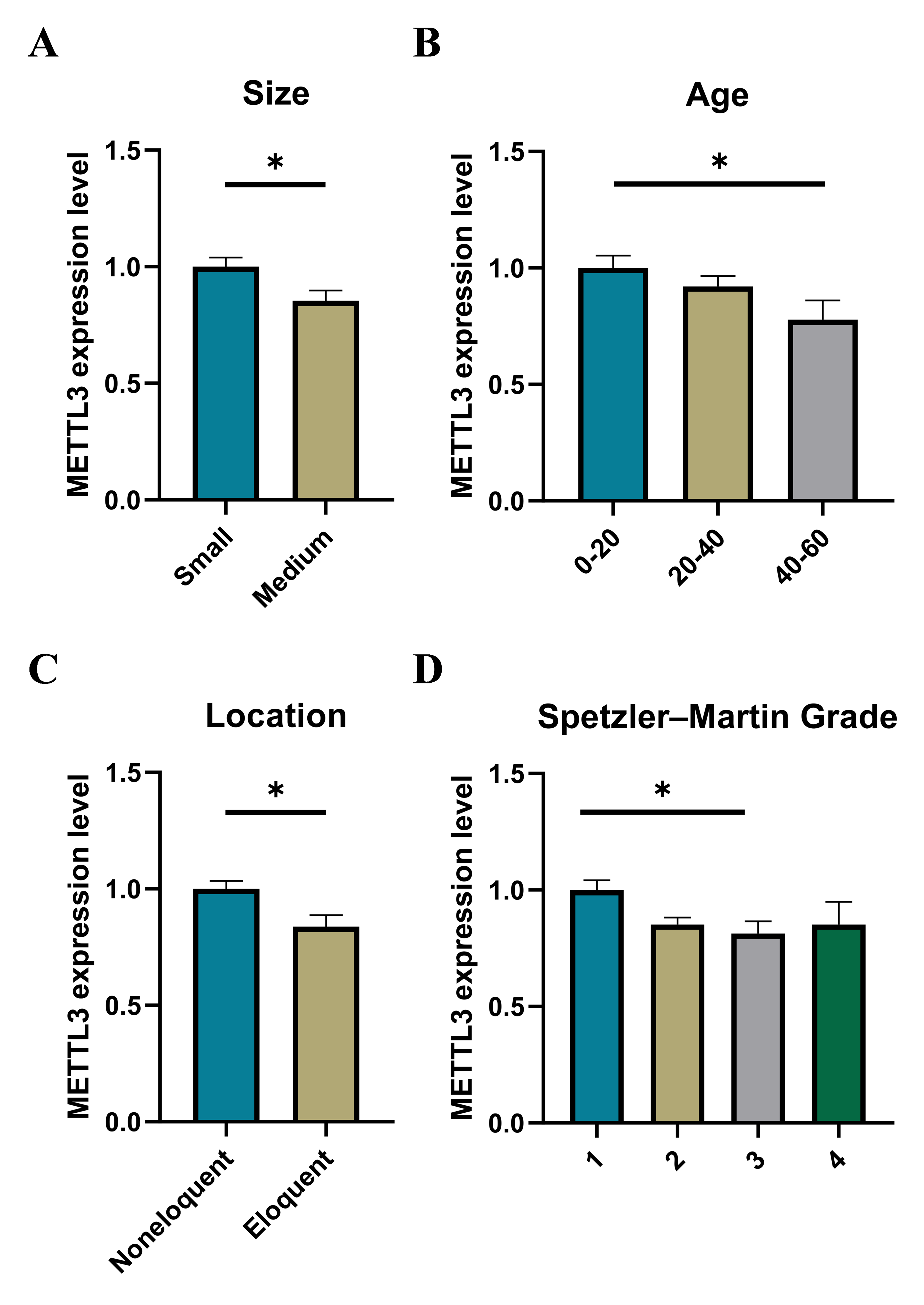

Supplement: Supplementary file 5 — Additional file 5: Figure S1. The expression levels of METTL3 in different groups of bleeding cerebral AVMs. (A) The expression levels of METTL3 in different size groups of bleeding cerebral AVMs. Small, 0 cm < MD ≤ 3 cm, n = 10; Medium, 3 cm < MD ≤ 6 cm, n = 11. (B) The expression levels of METTL3 in different age groups of bleeding cerebral AVMs. 0 < age ≤ 20, n = 10; 20 < age ≤ 40, n = 7; 40 < age ≤ 60, n = 4. (C) The expression levels of METTL3 in different location groups of bleeding cerebral AVMs. Eloquent, n = 11; Noneloquent, n = 10. (D) The expression levels of METTL3 in different Spetzler-Martin grading groups of bleeding cerebral AVMs. Grade 1, n = 7; Grade 2, n = 3; Grade 3, n = 7; Grade 4, n = 4. P values were calculated using one-way ANOVA and/or Student’s t-test. *, P < 0.05; **, P < 0.01; ***, P < 0.001. [file 12929_2020_655_MOESM5_ESM.tif]

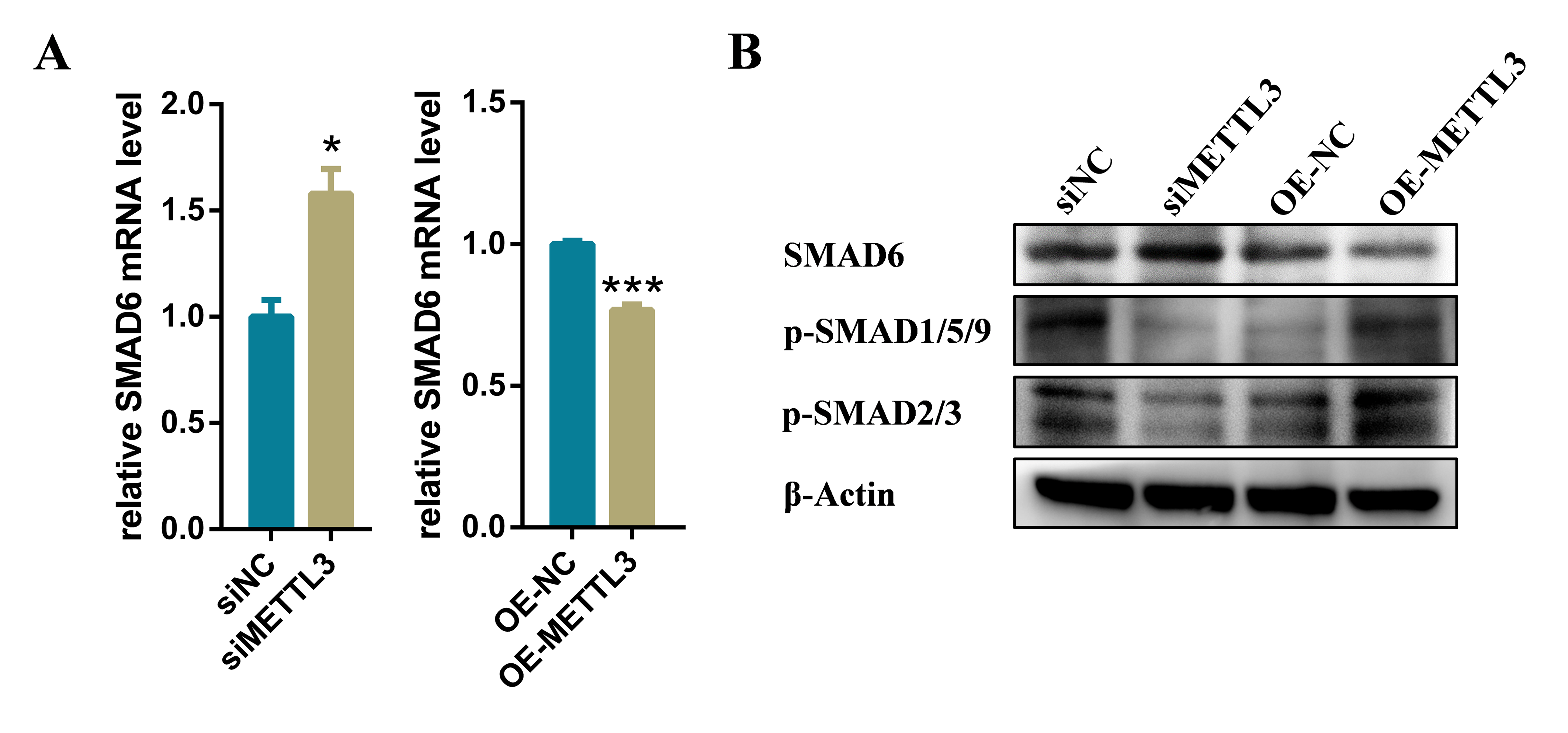

Supplement: Supplementary file 6 — Additional file 6: Figure S2. SMAD6 is up-regulated in METTL3 deficient endothelial cells. (A) qRT-PCR and (B) western blot analysis of the expression level of indicated genes in METTL3 silencing cells. Data are shown as mean ± SEM of three independent experiments. P values were calculated using Student’s t-test. *, P < 0.05; **, P < 0.01; ***, P < 0.001. [file 12929_2020_655_MOESM6_ESM.tif]

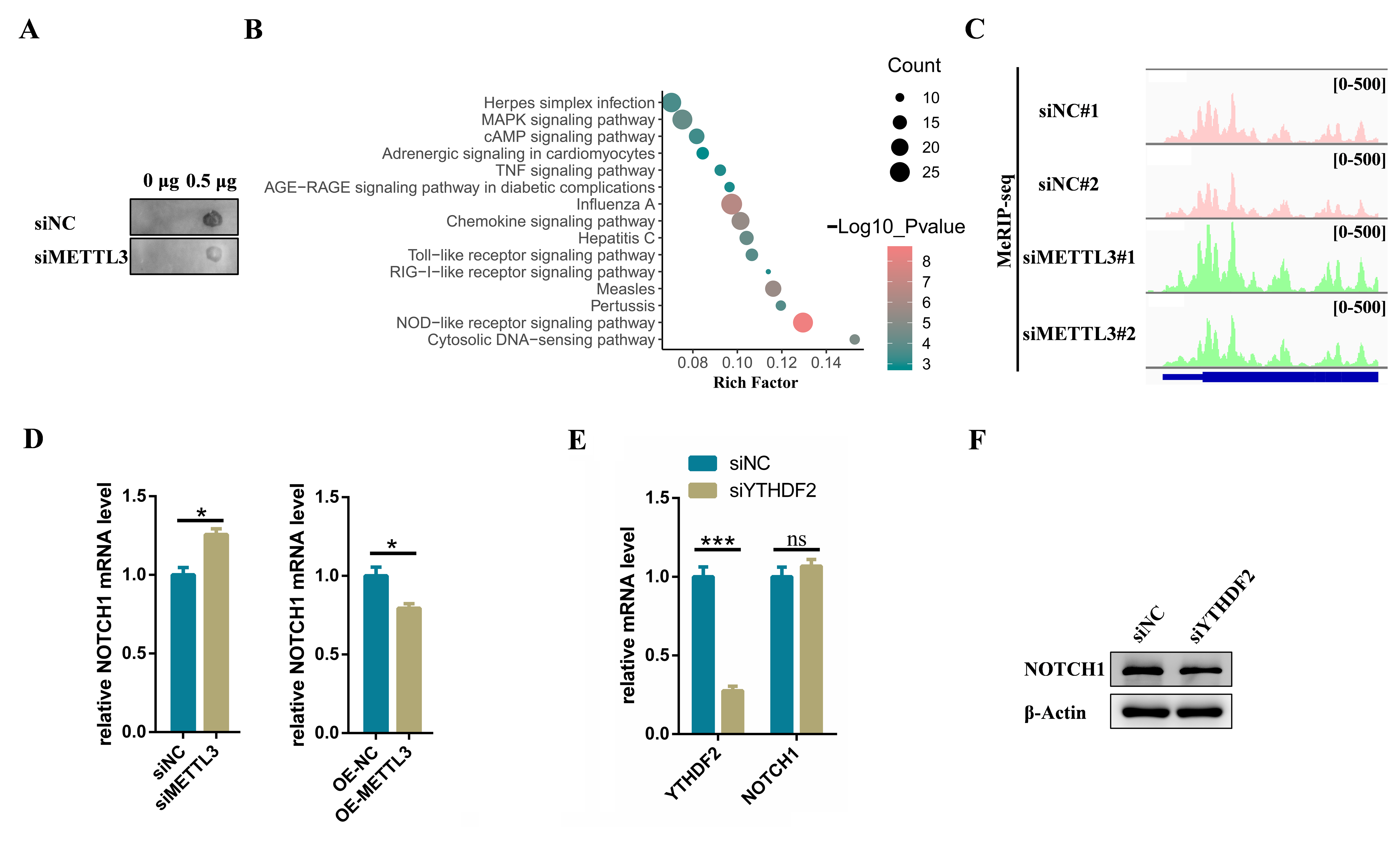

Supplement: Supplementary file 7 — Additional file 7: Figure S3. Knockdown of METTL3 does not reduce m6A enrichment in Notch1 mRNA. (A) Dot blot showing the m6A modification level in control and METTL3 deficient endothelial cells. (B) GO analysis of the low m6A modification genes in METTL3 knockdown endothelial cells. (C) Integrative Genomics Viewer (IGV) tracks displaying MeRIP-seq read distribution in Notch1 mRNA of control and METTL3 knockdown endothelial cells. (D) qRT-PCR analysis of the expression level of Notch1 after silencing or overexpressing METTL3. (E) qRT-PCR and (F) western blot analysis of the expression level of Notch1 in YTHDF2 silencing cells. Data are shown as mean ± SEM of three independent experiments. P values were calculated using Student’s t-test. *, P < 0.05; **, P < 0.01; ***, P < 0.001. [file 12929_2020_655_MOESM7_ESM.tif]

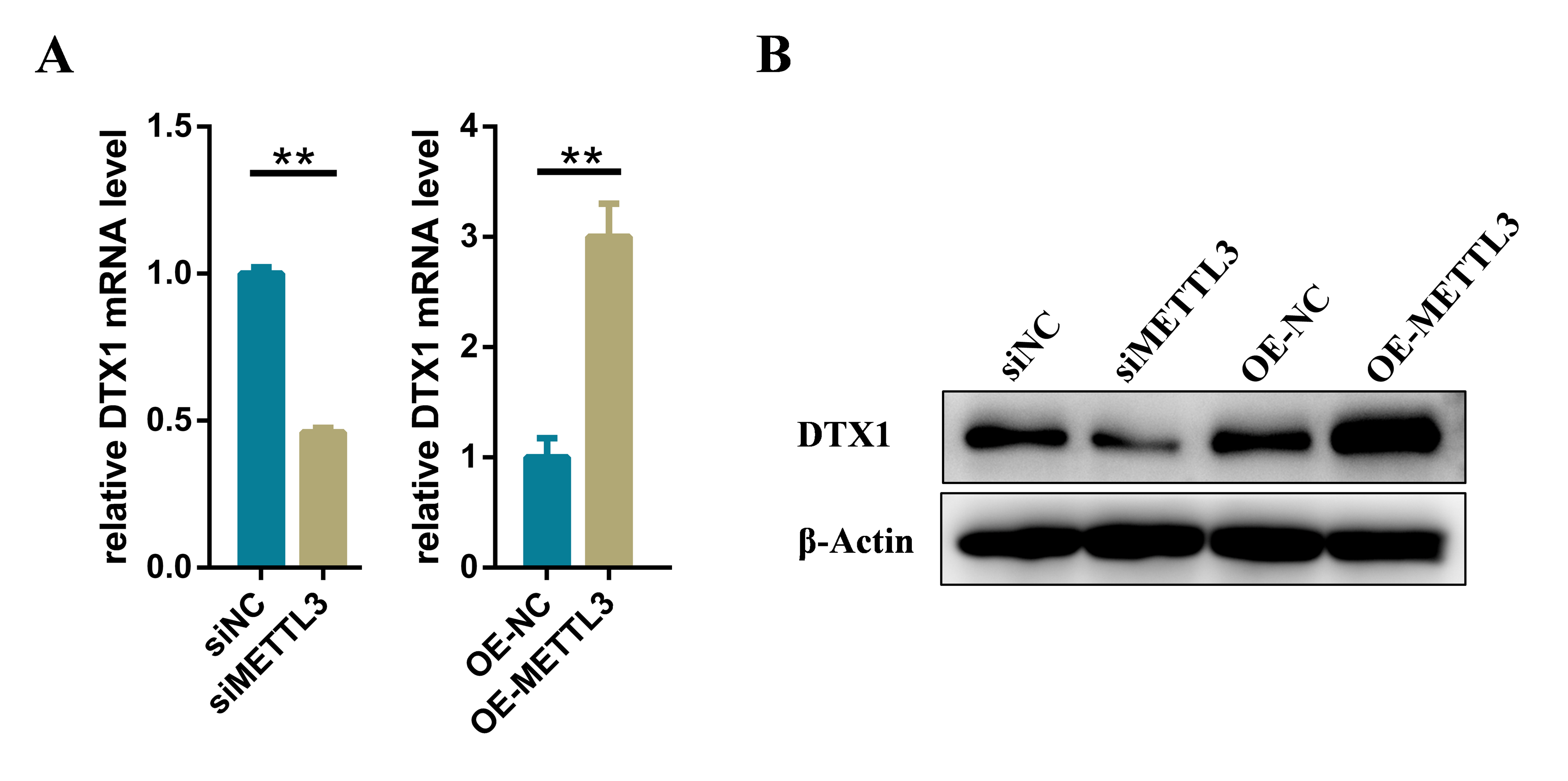

Supplement: Supplementary file 8 — Additional file 8: Figure S4. DTX1 is regulated by METTL3 in endothelial cells. (A) qRT-PCR and (B) western blot analysis of the expression level of DTX1 in METTL3 knockdown endothelial cells. Data are shown as mean ± SEM of three independent experiments. P values were calculated using Student’s t-test. *, P < 0.05; **, P < 0.01; ***, P < 0.001. [file 12929_2020_655_MOESM8_ESM.tif]
